# Inguinal Bladder Hernia Indirectly Treated With Prostate Artery Embolization

**DOI:** 10.7759/cureus.43090

**Published:** 2023-08-07

**Authors:** Alec Garfinkel, Ashita Tanwar, Michael C Larson

**Affiliations:** 1 Radiology, HCA Florida Brandon Hospital, Tampa, USA; 2 Radiology, California Northstate University College of Medicine, Elk Grove, USA; 3 Radiology, University of California, Davis, Sacramento, USA

**Keywords:** inguinal hernias, inguinal hernia repair, bladder diverticulum, urinary catheterization, lower urinary tract symptoms, urinary obstruction, recurrent hematuria, benign prostatic hyperplasia, prostate artery embolization, inguinal bladder hernia

## Abstract

An inguinal bladder hernia (IBH) is an abnormal protrusion of the bladder into the inguinal canal accompanied by a peritoneum sheath that creates the hernia sac. Clinical presentations vary greatly from lower urinary tract symptoms (LUTS) and reduction in scrotal size after voiding to being entirely asymptomatic. Since inguinal bladder hernias are uncommon and often accompanied by varied and nonspecific symptoms, it is challenging to diagnose and rarely included in differentials. Currently, computerized tomography (CT) imaging with contrast or voiding cystourethrography is recommended for diagnosis. There is no consensus on the best treatment for inguinal bladder hernias, with options ranging from laparoscopic repair to catheterization. In this study, we report the case of inguinal bladder hernia in an 86-year-old male presenting with symptoms of recurrent hematuria and two failed voiding trials after a Foley catheter placement from prostatomegaly resulting in bladder diverticula, and IBH. He was treated with prostate artery embolization (PAE) to address LUTS related to benign prostatic hyperplasia (BPH). The resultant decreased prostatic volume resolved his symptoms of IBH, hematuria, and urinary retention.

## Introduction

Inguinal bladder hernia (IBH), occasionally referred to as an inguinovesicular hernia, is an uncommon diagnosis involving the bladder protruding into an inguinal hernia sac. This often occurs due to an acquired peritoneal defect, which allows for herniation of bladder tissue into the inguinal canal [1]. Fewer than 4% of all inguinal hernias are reported to involve the bladder, with incidence increasing to 10% in obese men over the age of 50 [2]. Patients with this condition may present with a scrotal mass and nonspecific symptoms associated with bladder obstruction, such as hematuria, urinary urgency, and double voiding; however, many patients are asymptomatic and diagnosed intraoperatively during herniorrhaphy or incidentally via radiology studies [3]. Differential diagnoses for these symptoms may include other hernia, lymphadenopathy, femoral artery aneurysm, and pelvic malignancy [4]. Early diagnosis and treatment of inguinal bladder herniation are encouraged to prevent severe urological sequelae, including urinary tract infections, bladder ischemia and infarctions, and postoperative complications [2]. Common treatments for inguinal bladder herniation are resection or reduction of the bladder to its original location during open or laparoscopic herniorrhaphy or, more conservatively, catheterization of the urethra [1]. 

We present the case of an 86-year-old male with a history of recurrent hematuria and lower urinary tract symptoms (LUTS) due to prostatomegaly/benign prostatic hypertrophy (BPH), atrial fibrillation, aortic aneurysm, carotid artery stenosis, and ischemic stroke diagnosed with IBH. Surgical treatment of his urinary issues was not an option for this patient and urethral catheterization was suboptimal. Subsequently, the patient underwent prostate artery embolization to treat benign prostatic hyperplasia (BPH), which alleviated the patient’s urinary tract symptoms [5].

## Case presentation

An 86-year-old man presented to the emergency department (ED) with recurrent hematuria and a medical history significant for atrial fibrillation, aortic aneurysm, carotid artery stenosis, and ischemic stroke. The patient also endorsed occasional urinary retention, which began a few years ago and progressively worsened. The patient denied any fevers, chills, fatigue, weakness, lightheadedness, weight loss, dyspnea, cough, nausea, vomiting, dysuria, or any palpable pain.

The patient initially had hematuria a few years before presenting to the emergency department. He had been on a direct oral anticoagulant (DOAC) given his history of atrial fibrillation and stroke and had brought up minor hematuria to his healthcare team but decided to seek emergency medical care in this case due to the larger amount than normal. There were no other significant medical conditions (including coagulability disorders) or social factors. The patient was retired, lived at home with his wife, and did not smoke or drink alcohol. He denied any recreational drug use and was compliant with his current medication regime.

**Figure 1:**
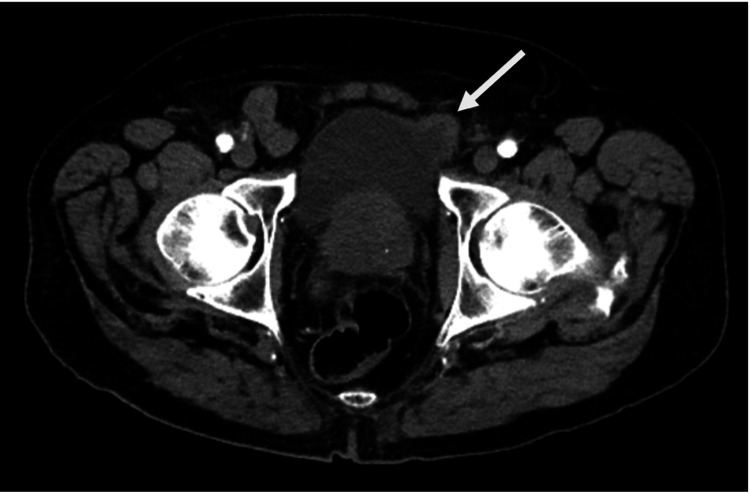
Axial arterial-phase CT scan through the lower pelvis showing a left anterior bladder bulge (white arrow) CT: computed tomography

Physical examination at the time was unremarkable. Although his complete blood count, basic metabolic panel, and renal function tests were within normal limits, an axial arterial computerized tomography (CT) scan revealed a left anterior bladder bulge with some bladder wall thickening (Figure 1). He was discharged from the ED and scheduled to follow up with urology.

**Figure 2:**
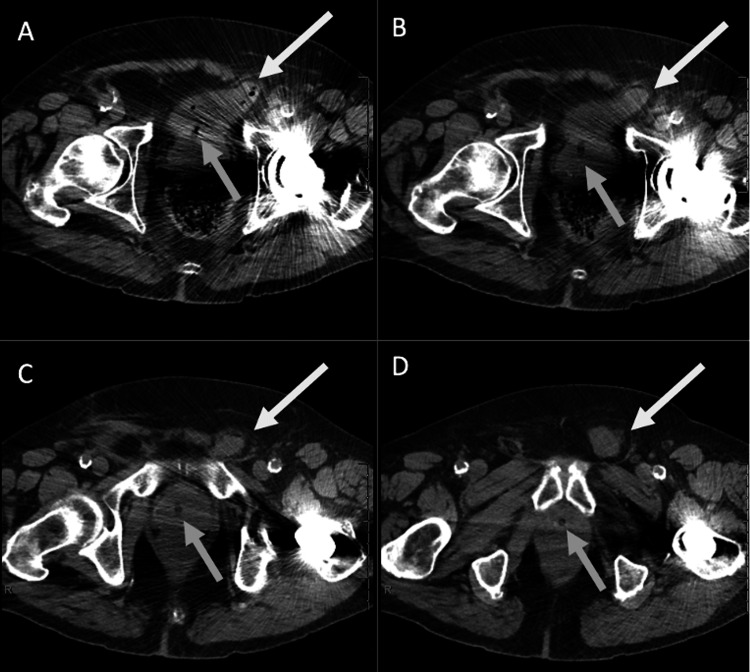
The axial non-contrast CT portion of a urogram as part of hematuria workup, progressing superiorly (A) through the level of the femoral heads (B & C), and inferiorly (D) to the lower pelvis shows a left anterior bladder bulge containing fluid and gas locules that extend into the left inguinal canal (white arrows) The Foley catheter can be faintly seen (gray arrow). A total left hip arthroplasty results in streak artifact across all images at these levels.

**Figure 3:**
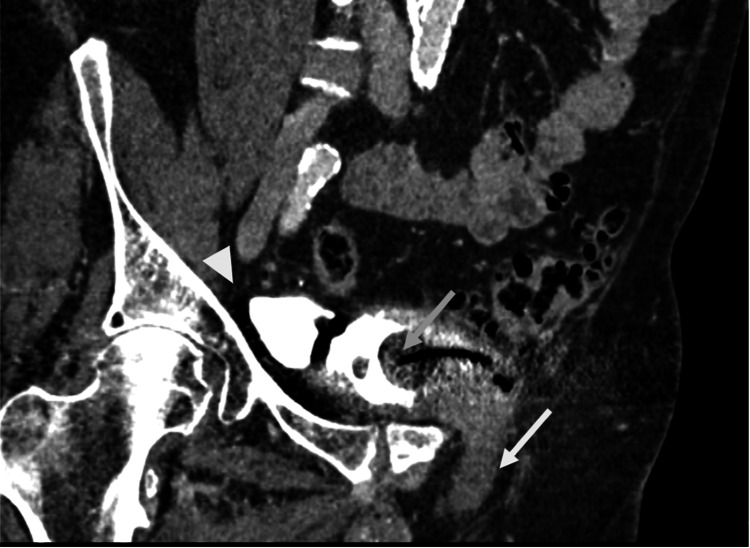
Oblique coronal reformats of the pelvis on CT urogram show a Foley catheter balloon in the bladder (gray arrow), a bladder diverticulum filled with contrast on the patient’s right (white arrowhead), and the urine-filled bladder hernia extending into the left inguinal canal (white arrow)

Further evaluation by urology revealed a symmetric and enlarged non-tender prostate with a smooth consistency and no palpable hard lumps. No gross blood, hemorrhoids, or fissures were identified. Additional lab work (prothrombin (PT), and international normalized ratio (PT-INR), urine culture) were all within normal limits. He was noted to have significant urinary retention, so a Foley catheter was placed. As part of his hematuria workup, he had a CT urogram, which identified a bladder bulge in the left inguinal canal that appeared to be causing an inguinal bladder hernia on the initial non-contrast portion (Figure 2). In the urogram phase of the study, the contrasted urine did not make it into the inguinal bladder hernia (IBH) but demonstrated a clear bladder diverticulum (Figure 3).

**Figure 4:**
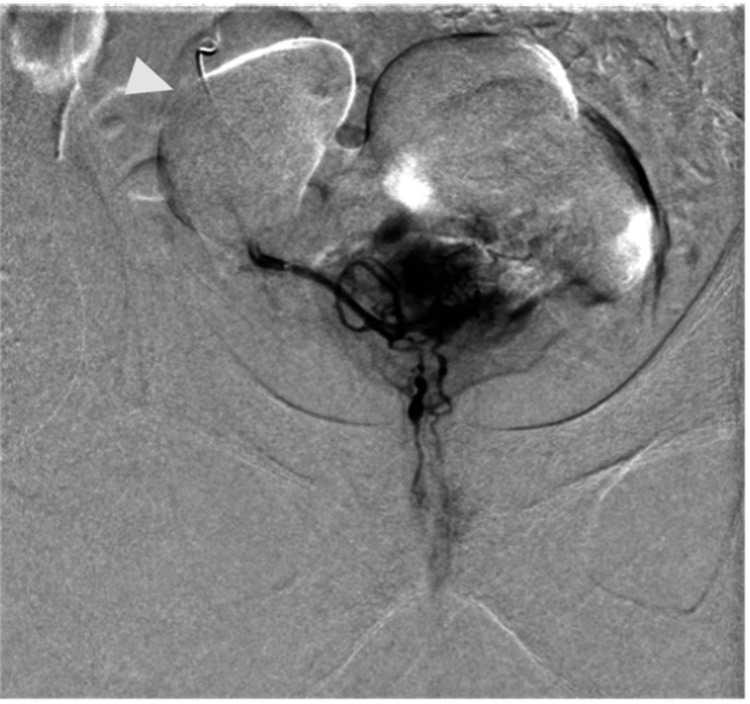
DSA of the right prostate artery before embolization shows typical enhancement of the central prostate gland A faint outline of the right bladder diverticulum can be seen (white arrowhead). DSA: digital subtraction angiography

**Figure 5:**
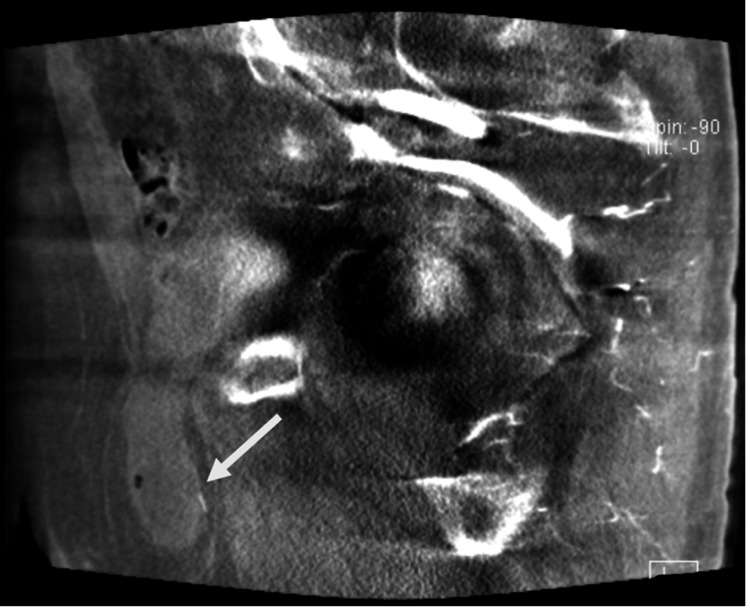
Sagittal reformats from an intraprocedural spin CT during prostate artery embolization show the bladder hernia extending into the inguinal canal CT: computed tomography

He was placed on Proscar (finasteride) 5 mg per oral daily with a scheduled follow-up appointment with urology. During the follow-up urology visit, the patient, unfortunately, failed his voiding trial again. Thus, his catheter was replaced and continued indefinitely. The patient underwent a cystoscopy, which revealed prostatomegaly and bladder diverticula. Following cystoscopy and the placement of a new Foley catheter, the urologist recommended referral to endourology to discuss possible holmium laser enucleation of the prostate (HoLEP) procedure. The patient was not considered a good surgical candidate given his cardiovascular history. He was referred to interventional radiology for consultation and the decision was made to proceed with prostate artery embolization (PAE) to alleviate symptoms from the patient’s chronic lower urinary tract outlet obstruction (LUTO). Digital subtraction angiography (DSA) was performed before embolization to localize the targeted right prostate artery (Figure 4). Additionally, images were generated intraprocedurally with a three-dimensional (3D) C-arm redemonstrating the IBH during PAE (Figure 5).

Postprocedurally, the patient resumed a normal diet and activities without issues. A few weeks later, at his outpatient follow-up visit, the patient successfully passed his voiding trial, and his Foley catheter was removed. At the nine-month follow-up visit, the patient reported resolution of his presenting symptoms, including no more hematuria or urinary retention. The patient felt pain-free and mentioned that his groin soreness resolved. The patient acknowledged the interventional radiologist and expressed how happy he felt with the outcome of the PAE. He has since discontinued Proscar (finasteride) for benign prostatic hyperplasia (BPH).

## Discussion

Inguinal hernias are the most common type of hernia, affecting 25% of all adults assigned male at birth [6]. Some hernias may be asymptomatic or cause mild discomfort while others can become painful and require surgical intervention. This is especially the case for inguinal bladder hernias, which comprise approximately 1-3% of inguinal hernias [7]. IBHs range in severity depending on the extent of bladder involvement. Larger IBHs may even involve the ureter, which can progress to obstruction and renal failure if left untreated. The size of the bladder herniation is proportional to the frequency of lower urinary tract symptoms (LUTS), including increased urinary frequency, urinary urgency, urinary hesitancy, straining, incontinence, and weak urinary stream [4]. Patients with this condition can be difficult to diagnose because they are either asymptomatic or present with nonspecific symptoms that often overlap with other pathological processes, including cysts, infection, stone obstruction, malignancy, congenital abnormality, neuropathy, and inflammatory conditions such as interstitial cystitis [4]. However, the physical exam can help narrow the differential diagnoses by examining the patient both standing and lying down to evaluate for a palpable bulge. If no bulge is palpated, having the patient cough can elicit a cough impulse, which is a useful sign suggestive of a hernia [6]. Regarding IBHs, noticing a groin protuberance shrink after void is helpful for diagnosis. Specifically, some patients may present with Mery’s sign, where manual compression of the bladder is required for voiding [4]. This finding should prompt further imaging to assess the characteristics of the hernia and confirm the underlying etiology.

IBHs occur mostly in males because men generally have larger inguinal canals compared to women [4]. Additionally, men tend to have weaker abdominal muscles and connective tissues than women. As men age, the fibromuscular abdominal wall tissues surrounding the inguinal canal weaken. If intra-abdominal pressure exceeds the counteractive force of the abdominal wall, the abdominal wall is likely to dismantle at its weakest point over time resulting in herniation. Unfortunately, this happened to be the case with our patient. Benign prostatic hyperplasia (BPH) often leads to chronic outflow obstruction, which inevitably leads to urinary retention and unwarranted bladder pressure. In conjunction with a weakened abdominal wall, chronic bladder outlet obstruction weakens the bladder wall increasing the likelihood of complications such as diverticulum with or without herniation [8]. Other risk factors for inguinal bladder hernia include chronic obstructive pulmonary disease (COPD), smoking, obesity, pelvic floor weakness, vasculopathy, and positive family history [4]. Occasionally, IBHs can stem from urologic malignancy. A study from 2004 showed that approximately 11.2% of IBHs were associated with urologic malignancies [8]. Thus, if the bladder is found within the contents of an inguinal hernia sac urology should be consulted. 

While a thorough history and focused physical examination are usually sufficient to diagnose an inguinal hernia, IBHs often require a more in-depth inquiry. This is especially the case when patients endorse recurrent hematuria and urinary retention spanning over a few years. Ultrasonography (US) is usually the first-line imaging modality due to its convenience, accessibility, and efficiency relative to CT or magnetic resonance imaging (MRI). Ultrasonography of an inguinal bladder hernia may demonstrate a hypoechogenic or anechoic mass lesion protruding from the bladder through the inguinal canal into the scrotum [8]. However, if a hernia is suspected in patients without a palpable hernia or in obese males over 50 years old presenting with LUTS, a CT scan or MRI followed by cystoscopy is generally advised to confirm the diagnosis and rule out herniation through uncommon sites as well as concurrent medical conditions [8]. Common findings on MRI and CT include an asymmetric bladder with a visible smooth outpouching toward the side of the hernia [7]. If ultrasonography, CT, or MRI findings are inconclusive, voiding cystourethrography is recommended [2]. It is considered to be the gold standard for diagnosing IBH, often showing a dog-ear or dumbbell shape of the bladder [2]. In our case, the CT scan revealed a left anterior bladder bulge that eventually progressed to involve the inguinal canal. Although this confirmed the diagnosis, a CT urogram and cystoscopy were completed to further determine any abnormalities within the bladder as well as throughout the urinary tract. Cystoscopy confirmed prostatomegaly, validating the patient’s IBH and chronic LUTS and the likely etiology for the bladder diverticulum and IBH. This proved to be helpful, as the patient was appropriately referred to a specialist for intervention.

Patients diagnosed with IBH are usually given the option of conservative management or surgical repair. The recommendation usually depends on the size of the hernia, the patient’s sex, and whether the patient is experiencing pain as well as difficulties with voiding. For small, asymptomatic hernias, watchful waiting is generally the plan with avoidance of activities that are prone to increasing intra-abdominal pressure [9]. If the patient’s hernia enlarges or the patient becomes symptomatic, surgical treatment is indicated [9]. The goals of surgery are to reposition the herniated bladder to its normal anatomic position and reinforce the abdominal wall defect with either a synthetic mesh or autologous tissue. Depending on the surgeon’s preference, the patient’s clinical presentation, and the characteristics of the hernia, a carefully planned decision is made to repair the IBH with either an open or laparoscopic approach. Both techniques have their benefits and risks, but surgeons generally recommend laparoscopic repair, as it has proven to shorten hospital stays, expedite patient recovery, and reduce the risks of postoperative complications. Large body habitus as well as complex, incarcerated, strangulated, or recurrent hernias are situations in which an open approach may be recommended [9]. If the herniated bladder wall is incarcerated or necrosed, bladder resection may be indicated during surgery [2].

The case presented here is unusual because, despite the patient not being a viable candidate for surgery, he was still able to receive life-altering treatment with PAE to alleviate his chronic LUTS and indirectly reduce his IBH. When compared with more invasive surgical procedures for BPH, such as transurethral resection of the prostate (TURP), PAE has a lower risk of urinary incontinence and postoperative sexual side effects [10]. For patients that pursue PAE, favorable outcomes have been reported for 75-80% of men [11]. Although this is a relatively new procedure, PAE provides long-term outcomes with results lasting as long as three to four years [11]. However, its widespread adoption for the treatment of BPH is still not widely accepted, as researchers and clinicians are still evaluating its long-term efficacy, safety, selection criteria, and embolization technique.

## Conclusions

Patients with IBH can present very differently from one another and can be difficult to diagnose clinically. It may be observed in asymptomatic patients, incidentally discovered during surgery, or revealed as the underlying pathology in patients presenting with nonspecific symptoms through imaging and physical examination findings. While a complete history and physical examination can be enough to diagnose IBH, imaging is often still recommended to evaluate the contents of the hernia as well as the underlying etiology, associated complications, and additional anatomic information helpful for surgical planning. IBHs are either treated conservatively or surgically repaired. PAE is an emerging technique for BPH and can potentially be a standard treatment option for candidates ineligible for surgery that are experiencing symptomatic bladder outlet obstruction.
